# Exploring green environmental composites as hosts for shielding materials using experimental, theoretical and Geant4 simulation methods

**DOI:** 10.1038/s41598-024-68028-z

**Published:** 2024-08-05

**Authors:** Mahmoud T. Alabsy, Mahmoud I. Abbas, Mahmoud A. Sharaby, Mohamed Abd Elzaher, A. S. Doma, Ahmed M. El-Khatib

**Affiliations:** 1 Physics Department, Faculty of Science, Alexandria University, Alexandria, 21511 Egypt; 2 Department of Basic and Applied Science, Faculty of Engineering, Arab Academy for Science, Technology, P.O 1129, El Alamein, Egypt; 3 Advanced Technology and New Materials Research Institute (ATNMRI), City of Scientific Research and Technological Applications (SRTA-City), New Borg Al-Arab City, 21934 Alexandria Egypt

**Keywords:** Rice straw composite, Animal glue, PVA glue, Gamma-ray shielding, Geant4 simulation, Attenuation parameters, Nuclear physics, Composites

## Abstract

Rice straw is considered an agricultural waste harmful to the environment, which is abundant in most parts of the world. From this point, the present study is devoted to preparing new composites of two types of glue based on rice straw as a plentiful, low-cost matrix. Straw glue samples were prepared by mixing 20% wt. of rice straw with 80% wt. of animal glue (RS-An) and polyvinyl acetate (RS-PVA_C_) at different thicknesses of 1, 2, and 3 cm. The chemical composition of the prepared samples was identified by energy dispersive X-ray analysis and their morphology was examined using a scanning electron microscope. The mechanical test explored that RS-An and RS-PVA_C_ respectively required a stress of 25.2 and 25.5 MPa before reaching the breaking point. γ-ray shielding performance was analyzed and determined at numerous photon energies from 0.059 to 1.408 MeV emitted from five-point γ-rays sources using NaI (Tl). Linear attenuation coefficient was calculated by obtaining the area under the peak of the energy spectrum observed from Genie 2000 software in the presence and absence of the sample. The experimental results of mass attenuation coefficient were compared with theoretical data of XCOM software with relative deviation ranging from 0.10 to 2.99%. Geant4 Monte Carlo simulation code was also employed to validate the experimental results. The relative deviation of XCOM and Geant4 outcomes was 0.09–1.77%, which indicates a good agreement between them. Other radiation shielding parameters such as half value layer (HVL), tenth value layer, and mean free path were calculated in three ways: experimentally, theoretically from the XCOM database, and by simulation using Geant4 code. Additionally, effective atomic number (Z_eff_), effective atomic number (N_eff_), equivalent atomic number (Z_eq_), and buildup factors were evaluated. It was confirmed that the γ-ray shielding properties were further boosted by mixing rice straw with the animal glue compared to the synthetic one.

## Introduction

Humans are exposed to ionizing radiation from a variety of sources throughout their lives, including natural radiation sources, medical applications, industrial applications, liquid nuclear waste, and the effects of nuclear accidents^1^. Higher doses of ionizing radiation can harm people and damage biological tissues and DNA^2^. Radiation protection’s fundamental principle is to protect people and the environment from the harmful effects of ionizing radiation^3^. The three main ideas that can be applied in radiation protection are reducing the exposure time, extending the distance between the workers and the radioactive source, and using shielding materials. High-Z materials are known to have better gamma protection than lower-Z materials due to their higher probability of interacting with photons^4,5^. Compared with previous research, recent developments in polymer matrices containing high-Z and microscopic materials have improved their ability to absorb and reduce high-energy radiation. This is due to their geometrically shaped and lightweight nature, as well as their enhanced durability and flexibility. Additionally, these matrixes possess superior physical, mechanical, optical, and radiation resistance properties. Lately, plastic composites have offered promising, suitable alternatives to lead and concrete in the field of radiation shielding^6^. Moreover, it is considered the cornerstone of many applications, such as medical fields, civil engineering, aerospace technology, and electronics^7^.

Glue is one of the common polymeric materials. Glue has been in use since the beginning of civilization. Traditional types contain a colloidal solution in water, which sets after drying into a hard adhesive film. There are various types of glue materials, such as materials based on polyvinyl acetate (PVA_C_), which is made up of a water-based emulsion. It is also known commercially as wood glue, white glue, carpenter’s glue, school glue, or PVA glue^8^. On the other hand, animal glues are natural materials consisting largely of the protein collagen, which is extracted by boiling bones, hides, or horns^9^. Glues are usually nontoxic thermoplastic adhesives and are largely used in glass fiber-reinforced plastics to improve their stress and anti-shrink properties^10^. Glues are also used in automobile headlights to enhance their gloss performance. Furthermore, glues can be incorporated into cement and concrete composites to improve their water-resistance properties^11^.

Rice is a highly consumed crop in developing countries, with its residues being lightweight and biodegradable. It ranks second in global crop production, with a notable increase of 17.56 million tons per year over the past decade^12^. Rice straw is an abundant and eco-friendly resource, possessing the qualities of being natural, renewable, and reusable. Each year, a significant quantity of straw is produced globally, with China alone generating approximately 1 billion tons in 2019^13^. About 20–23% of the total rice comprises husk, which is readily available as waste material, and comes at no cost. This abundant resource contains 10–22% SiO_2_ in a hydrated amorphous form similar to silica gel, making it an excellent adsorbent^14^. Unfortunately, only a fraction of this straw is utilized, while the majority is either discarded or burned, resulting in severe environmental pollution and the squandering of valuable biomass resources. As a result, the production of residuals from rice will also increase by increasing the rice production rate, and then the pollution rate will also increase^15^.

Given their low density and excellent mechanical properties, rice straws are considered to be highly suitable fillers for the production of composite materials^16^. This type of composite material is used in place of plastic material. Natural fibers have good mechanical properties and are biodegradable^17^. This type of composite is used for house construction and agricultural items. Due to the various properties of rice straw, it can be reinforced as a filler material to be used in many industrial applications^18,19^. Taha et al. fabricated a new composite of nano rice straw/HDPE to achieve enhanced tribological performance. Also, they improved the lower friction coefficients, reduced wear, and enhanced mechanical properties by utilizing natural materials in the formulation of these bio-nanocomposites^20^. Megahed et al. conducted a study on rice straw and glass fiber-reinforced polyester composites, evaluating their mechanical, structural, and water absorption properties. The results of their comparison showed significant improvements in tensile strength, flexural strength, and other mechanical properties^21^. Ozturk et al.^22^ use the ash of rice husk instead of cement as a shielding material in prepared mortar at 10, 20, and 30 wt%. They concluded from mechanical and electromagnetic measurements that the mortars, including additives, can be used in various microwave engineering applications without sacrificing mechanical properties. In addition, El Kassas et al. present a new eco-friendly mechanical technique to prepare rice straw fibers, which are used as an alternative raw material for manufacturing medium-density fiberboard. They use no chemical pretreatment, and no costly drying process is required^23^. Furthermore, Huicheng et al. designed a new method for preparing rice straw-reinforced LLDPE composite by layering rice straw above LLDPE thin film, rolling it up, and then putting it under a hot press. This developed process presents a high mechanical property, improving thermal stability and better water resistance than the traditional method^24^. El-Sayed et al. mixed heavy concrete with recycled rice straw ash. The samples were exposed to gamma sources to determine the radiation shielding parameters. Results showed that concrete containing 15% rice straw ash is better for gamma-ray and neutron shielding^25^. Mostafa et al. manufactured a transparent glass from rice husk silica loaded with bismuth oxide to enhance its radiation protection properties. These measurements demonstrated that as Bi_2_O_3_ was increased, the gamma-ray mass attenuation coefficient (MAC) increased, and therefore, the half value layer (HVL) decreased. Among all glasses, the sample containing 20 mol% Bi_2_O_3_ was the best for attenuating 356 keV gamma-ray energy^26^.

After conducting an in-depth analysis of the existing literature, it became apparent that there needed to be more research studies that explored the use of rice straw composites as shielding materials against radiation. Therefore, our primary objective is to develop and fabricate ecologically sustainable materials that conform to the established radiation protection standards. Our overarching goal is to contribute to advancing innovative and environmentally responsible solutions in radiation protection. Therefore, in the current work, two new samples based on a mixture of rice straw, either with animal glue (RS-An) or polyvinyl acetate glue (RS-PVA_C_), were prepared. The composite blocks were prepared in various thicknesses to evaluate their effectiveness as shielding materials against radiation. The SEM and EDX analyses were identified for the prepared samples. The LACs of the investigated samples were calculated experimentally. In addition, various radiation shielding parameters were investigated, including MAC, HVL, TVL, MFP, Z_eff_, N_eff_, Z_eq_, and BFs. The XCOM database was also utilized to obtain the theoretical values of MACs. Moreover, the Geant4 toolkit was employed to simulate the radiation shielding parameters at various photon energies from 0.059 to 1.408 MeV and compared with the experimental and theoretical results.

## Materials and methods

### Material

Rice straw was collected from rice mills in some agricultural areas in Egypt. Then it was sieved to get rid of any impurities. The collected straw was crushed and ground into very small pieces by a blinder. Animal and white glue were imported from the local market.

### Sample preparation

**Figure 1:**
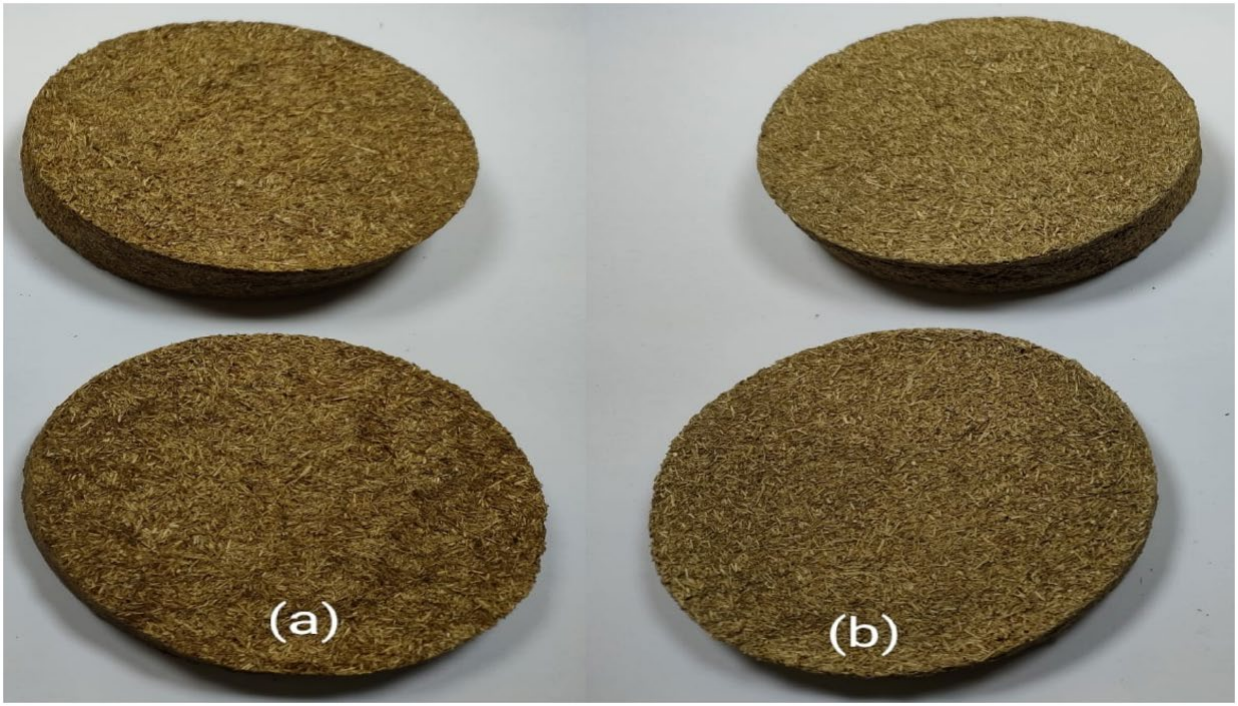
Prepared samples with various thicknesses: (**a**) RS-An and (**b**) RS-PVA_C_.

Straw-glue samples, shown in Fig. 1, were prepared by adding 20% straw to 80% glue material. The combination of straw and animal glue (RS-An) and straw and polyvinyl acetate (RS-PVA_C_) was thoroughly mixed until a uniform paste was achieved. This mixture was then poured into cylindrical molds with a diameter of 8 cm and varying heights of 1 cm, 2 cm, and 3 cm. The molds were left to dry naturally in the open air. This process ensures the formation of solid composite blocks with consistent composition and dimensions.

The Archimedes technique, according to ASTM D 792–9124, was applied to calculate the density of each composite sample by using Eq. (1) ^27^. For this purpose, a calibrated electrical balance with a single pan of accuracy of 0.0001 g and ethanol as an organic liquid was used.

1 $$\uprho = \frac{{\text{M}}}{{\text{V}}}$$

where M is the mass of the sample and V is its volume. The measured densities of RS-An and RS-PVA_C_ samples are 0.84767 and 0.73862 g/cm^3^, respectively 

### Morphology

The scanning electron microscope (SEM) analysis was conducted using the JSM-5300, JEOL type. The samples were coated using an ion sputtering coating device and then placed inside the electron microscope unit with an operating voltage of 20 keV, and the magnification order was 1000×.

### Mechanical measurements

**Figure 2:**
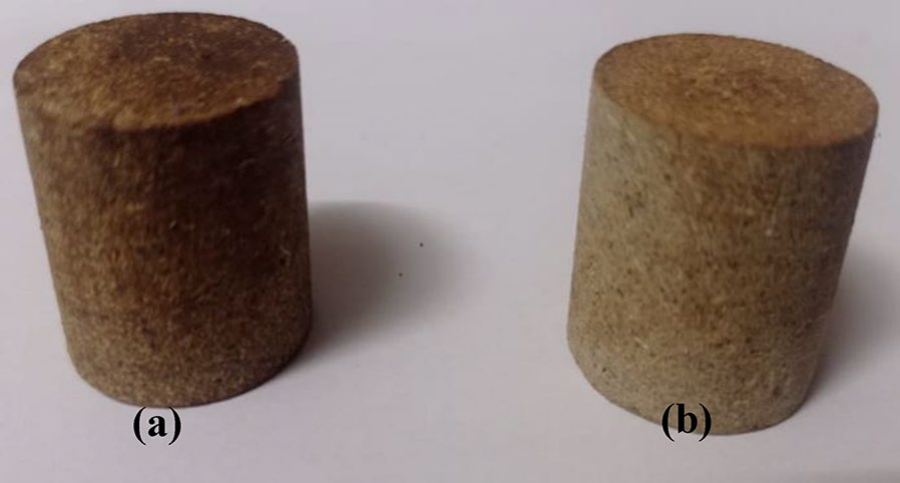
Rice straw/glue composites: (**a**) animal glue and (**b**) PVA_C_ glue for mechanical testing.

As shown in Fig. 2, the cylinder specimens of diameter 22 mm and thickness 22 mm were cast to determine compressive strength. The mechanical measurements were conducted at room temperature using the universal testing machine (UTM), and the compressive strength was measured at a rate of 50 mm/min.

### Gamma-ray spectroscopic setup

**Figure 3:**
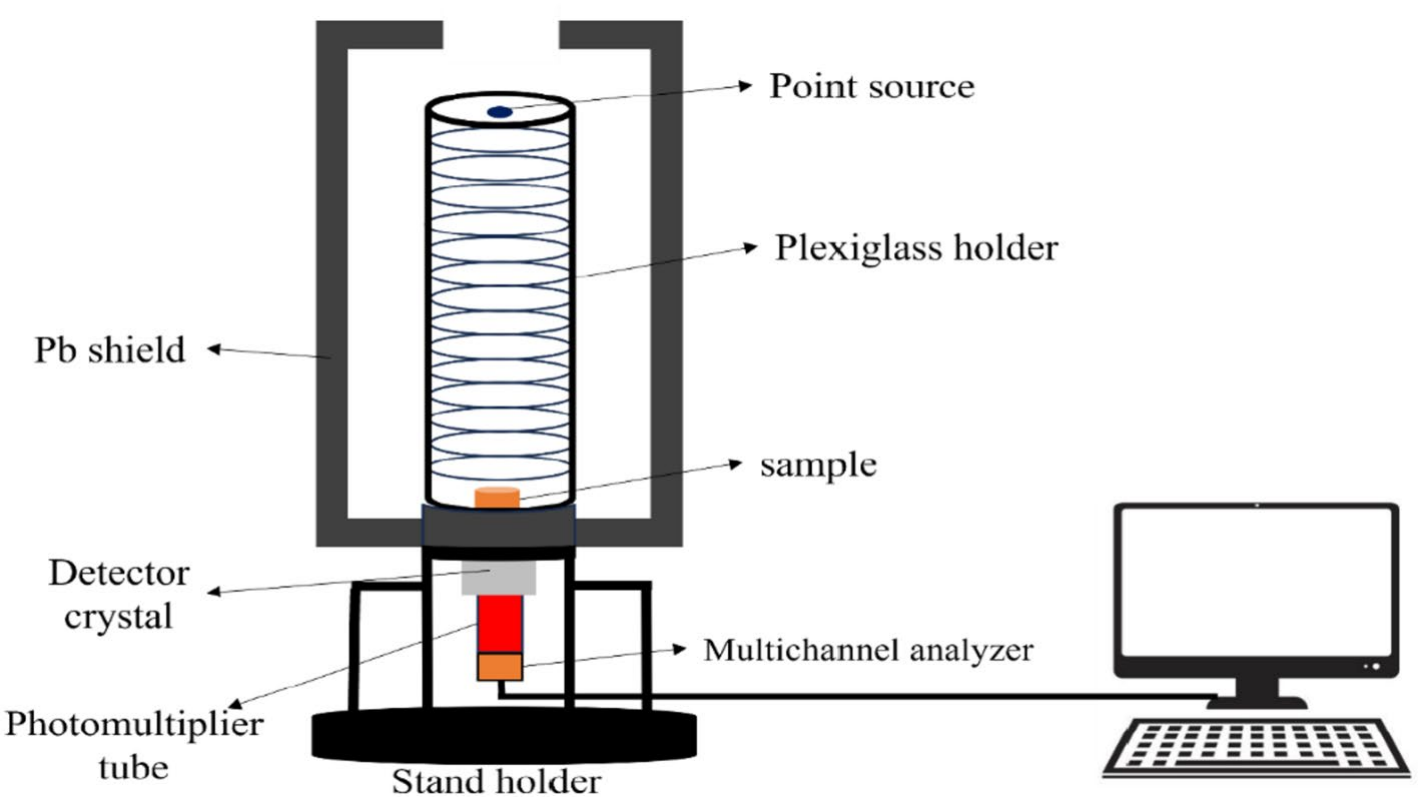
Schematic diagram of the NaI(Tl) scintillation detector.

**Figure 4:**
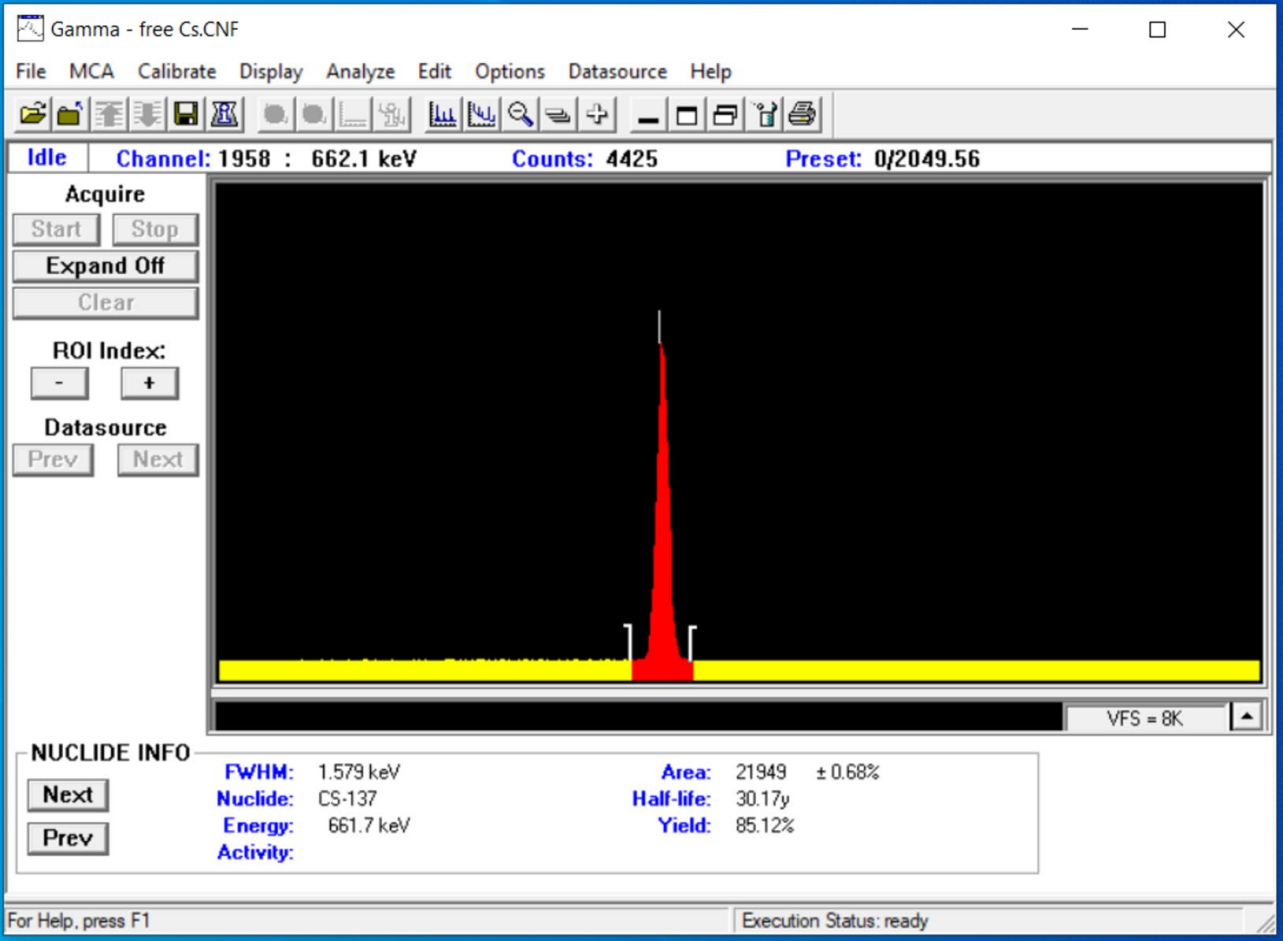
The acquired spectrum using Cs-137 radioactive source in the absence of absorbers.

The shielding parameters were calculated in an experiment that measured the intensity of the γ-rays passed through the specimen. The detector used in this work was a NaI(Tl) scintillation detector^28^. Figure 3 shows the configuration for this study, where the source-detector distance was 45 cm and the detector-sample distance was 4 cm. Five standard radioactive point sources are used as follows: Am-241, Co-60, Ba-133, Cs-137, and Eu-152 to emit a broad spectrum of energies ranging from 0.059 to 1.408 MeV, as listed in Table 1. The emitted photons interact with the detector crystal and transform into electrical signals that can be analyzed using Genie 2000 software. Figure 4 shows an example of the Cs-137 spectrum obtained in the absence of absorbers.

**Table 1:** Standard radioactive point sources and their emitted photon energies.

Radioactive sources	Photon energy (MeV)
Am-241	0.05953
Ba-133	0.08099
	0.35601
Cs-137	0.66166
Co-60	1.17323
	1.33250
Eu-152	0.12178
	0.24469
	0.34428
	0.77890
	0.96413
	1.40801

### Shielding parameters

The area under the photo-peak, related to used radioactive sources, was calculated in the presence and absence of the sample, which represents the transmitted (I) and initial (I_0_) intensities, respectively. Calculating the LAC is the first step to evaluating the material capability for shielding. LAC can be determined using Beer-Lambert’s law^29^:

2 $${\text{LAC}} = \frac{1}{{\text{t}}}{\text{Ln}}\left( {\frac{{{\text{I}}_{{\text{o}}} }}{{\text{I}}}} \right)$$

where t is the thickness of the absorber.

The MAC is measured by dividing the LAC for a given material by its density (ρ), as represented in Eq. (3) ^30^:

3 $${\text{MAC}} = \frac{{{\text{LAC}}}}{\rho }$$

The theoretical values of MACs for the given samples were calculated using XCOM software Code. Additionally, the relative deviation (Δ%) between the experimental and theoretical values of MACs at the investigated photon energies is listed in Table 2. The Δ% is evaluated according to the expression^31^:

4 $$\Delta (\% ) = \frac{{\left( {{\text{MAC}}} \right)_{{{\text{Xcom}}}} - \left( {{\text{MAC}}} \right)_{{{\text{Exp}}}} }}{{\left( {{\text{MAC}}} \right)_{{{\text{Xcom}}}} }} \times 100\%$$

**Table 2:** Chemical compositions of the prepend samples.

	RS-An	RS-PVA_C_
Elements	Weight fractions(wt.%)	
Density (g cm^−3^)	0.84767 ± 0.00775	0.73862 ± 0.018159
C	42.857	46.81
N	13.99	0
O	39.003	47.002
Na	0.5325	0.26
Mg	0.0775	0.11
Al	0.075	0.047
Si	1.8775	2.6
S	0.26	0.107
Cl	0.4275	0.177
K	0.3675	0.26
Ca	0.5325	2.627

The HVL is a crucial parameter when making an appropriate radiation-protecting substance. It is defined as the absorption thickness needed to decrease the incident radiation on the substance to 50% of its initial value and is calculated by Eq. (5) ^32^:

5 $${\text{HVL}} = \frac{\ln 2}{{{\text{LAC}}}}$$

Also, the thickness at which the gamma-ray photon intensity passes through the material decreases to a tenth of its initial value, called TVL, and is given by^33^:

6 $${\text{TVL}} = \frac{\ln 10}{{{\text{LAC}}}}$$

MFP is defined as the distance between two successive interactions; mathematically, it is the inverse of the LAC. The MFP was calculated for shielding material using the following Eq. (7) ^34^:

7 $${\text{MFP}} = \frac{1}{{{\text{LAC}}}}$$

The effect of the chemical composition of a shielding material is always clarified using the effective atomic number (Z_eff_), and its variation with energy may be used to investigate the relative changes in photon absorption processes with energy for various shields. Z_eff_ was calculated from MAC based on Eq. (8) ^35^:

8 $${\text{Z}}_{{{\text{eff}}}} = \frac{{\sum\nolimits_{{\text{i}}} {{\text{f}}_{{\text{i}}} {\text{A}}_{{\text{i}}} \left( {{\text{MAC}}} \right)_{{\text{i}}} } }}{{\sum\nolimits_{{\text{i}}} {{\text{f}}_{{\text{i}}} \frac{{{\text{A}}_{{\text{i}}} }}{{{\text{Z}}_{{\text{i}}} }}\left( {{\text{MAC}}} \right)_{{\text{i}}} } }}$$

where A_i_ and f_i_ are the atomic weight and the molar fraction of the i^th^ constituent element in the composite, respectively.

The effective electron density (N_eff_), measured in electrons/g, defined as the number of electrons per unit mass of the material, is derived using the calculated Z_eff_ according to Eq. (9) ^36^:

9 $${\text{N}}_{{{\text{eff}}}} = \frac{{{\text{N}}_{{\text{A}}} {\text{Z}}_{{{\text{eff}}}} }}{{\sum\nolimits_{{\text{i}}} {{\text{f}}_{{\text{i}}} {\text{A}}_{{\text{i}}} } }}$$

where $\sum\nolimits_{{\text{i}}} {{\text{f}}_{{\text{i}}} {\text{A}}_{{\text{i}}} }$ represents the mean atomic mass of the sample, and N_A_ is the Avogadro’s number.

A primary factor used for designing a radiation shielding medium is the buildup factor (BF). BFs are divided into two types: the energy absorption buildup factor (EABF) and the exposure buildup factor (EBF). BFs were obtained by computing them from the Geometrical Progression (G-P) fitting method at energies ranging from 0.015 to 15 MeV. To calculate BFs, firstly, it is important to compute the equivalent atomic number (Z_eq_) of the composite using Eq. (10) ^37^:

10 $${\text{Z}}_{{{\text{eq}}}} = \frac{{{\text{Z}}_{{1}} \log \frac{{{\text{R}}_{{2}} }}{{\text{R}}} + {\text{Z}}_{{2}} \log \frac{{\text{R}}}{{{\text{R}}_{{1}} }}}}{{\log \frac{{{\text{R}}_{{2}} }}{{{\text{R}}_{{1}} }}}}$$

where R is the ratio of MAC_Compton_/MAC_Total_ for the composite at a given energy. Also, Z_1_ and Z_2_ are atomic numbers of elements according to ratios R_1_ and R_2_, respectively. The calculated Z_eq_ values of the investigated materials were then used to interpolate the GP fitting parameters (b, c, a, X_K_, and d) in the range of energy between 0.015 and 15 MeV using the interpolation formula:

11 $${\text{C}} = \frac{{{\text{C}}_{{1}} \log \frac{{{\text{Z}}_{{2}} }}{{{\text{Z}}_{{{\text{eq}}}} }} + {\text{C}}_{{2}} \log \frac{{{\text{Z}}_{{{\text{eq}}}} }}{{{\text{Z}}_{{1}} }}}}{{\log \frac{{{\text{Z}}_{{2}} }}{{{\text{Z}}_{{1}} }}}}$$

where C_1_ and C_2_ are GP fitting parameters, taken from the ANSI/ANS-6.4.3 standard database^38^.

Finally, the BFs for the selected samples were then estimated with the help of the obtained GP fitting parameters using the following relations^39^_:_

12 $${\text{B}}({\text{E}},{\text{x}}) = 1 + \frac{{({\text{b}} - 1)({\text{K}}^{{\text{x}}} - 1)}}{{{\text{K}} - 1}}\,\,\,\,\,\,\,\,\,\,\,\,\,\,\,\,\,{\text{k}} \ne 1$$

13 $${\text{B}}({\text{E}},{\text{x}}) = 1 + ({\text{b}} - 1){\text{x}}\,\,\,\,\,\,\,\,\,\,\,\,\,\,\,\,\,\,\,\,\,\,\,\,\,\,\,\,\,\,\,\,{\text{k}} = 1$$

where K(E,x) is the photon dose multiplication factor, which is obtained by:

14 $${\text{K}}({\text{E}},{\text{x}}) = {\text{cx}}^{{\text{a}}} + {\text{d}}\frac{{\tanh \left( {\frac{{\text{x}}}{{{\text{x}}_{{\text{k}}} }} - 2} \right) - \tanh ( - 2)}}{1 - \tanh ( - 2)}$$

where x is the mean free path (MFP) that ranged from 1 to 40 mfp (1 ≤ x ≤ 40).

### Geant4 simulation

**Figure 5:**
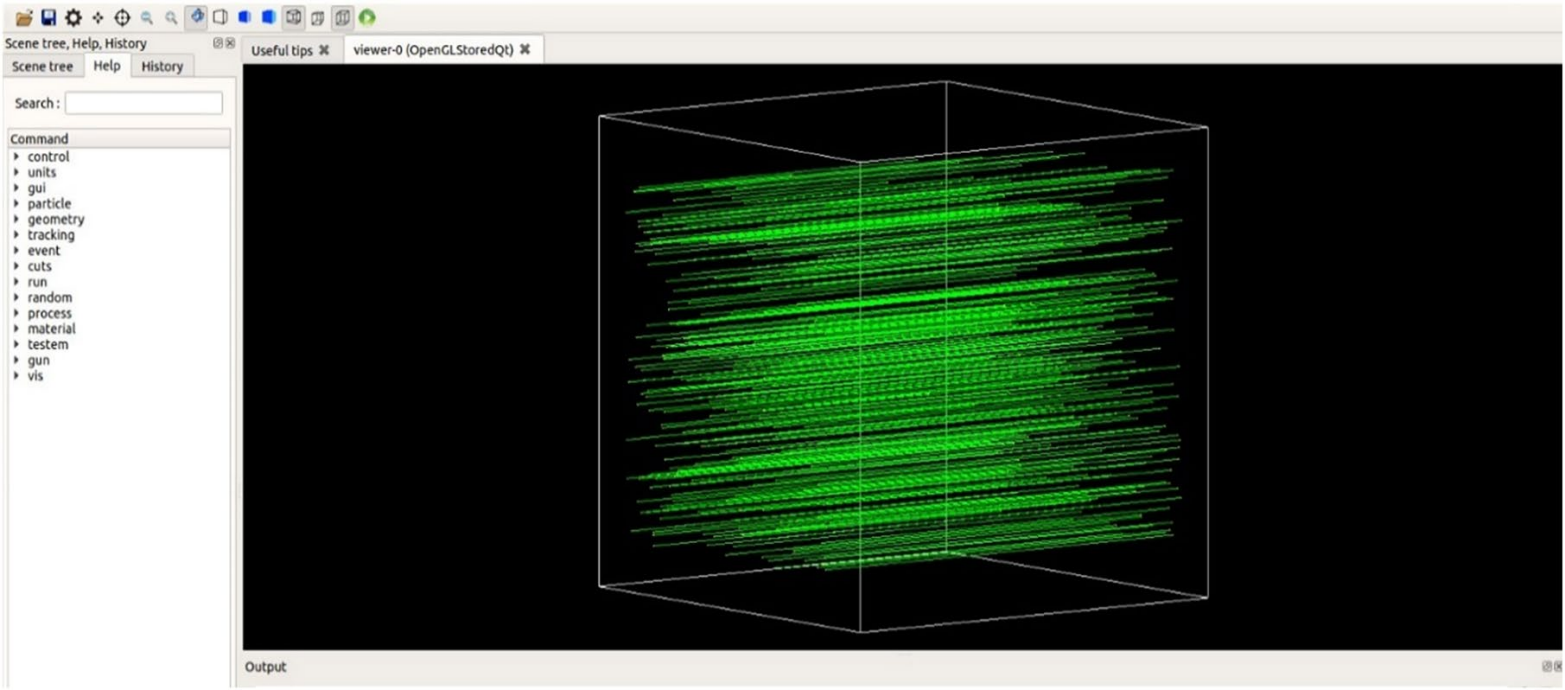
The Geant4 simulation setup.

Geant4 is a widely used object-oriented toolkit for simulating the transport of charged and neutral particles through matter^40^. Geant4 is based on Monte Carlo techniques, which are particularly well-suited for simulating complex particle interactions in matter^41^. In this study, we used the Geant4 toolkit to simulate the transport of photons through two different composite materials, RS-PVAc and RS-An, in the energy range of 0.059–1.408 MeV. The simulation aimed to measure the linear attenuation coefficients (LACs) of the two composites, from which other shielding parameters, including HVL, TVL, and MFP, can be calculated. The simulation was performed using Geant4 version 11 on a Linux operating system, using a single cubic box of side of 5 cm made of a homogenous material. Each composite sample was constructed in accordance with the component elemental weight fractions listed in Table 2. To simulate the LACs, we randomly projected 10^6^ main monoenergetic events, labeled as (I_0_), at the material’s edge as parallel rays. The Monte Carlo simulation uses this large number of incident events to minimize statistical error. During the simulation, the substance may either absorb or transmit every incident photon. The number of photons that dissipate energy due to the three primary interactions (photoelectric effect, Compton scattering, and pair production) and those transmitted in the absence of any interaction can be calculated at the end of the simulation. Using Eq. (2), LAC values for each composite can be determined, where (I) represents the total number of photons transmitted at the end of the simulation. The Geant4 simulation setup used in this study is illustrated in Fig. 5.

## Result and discussion

### Composition analysis

**Figure 6:**
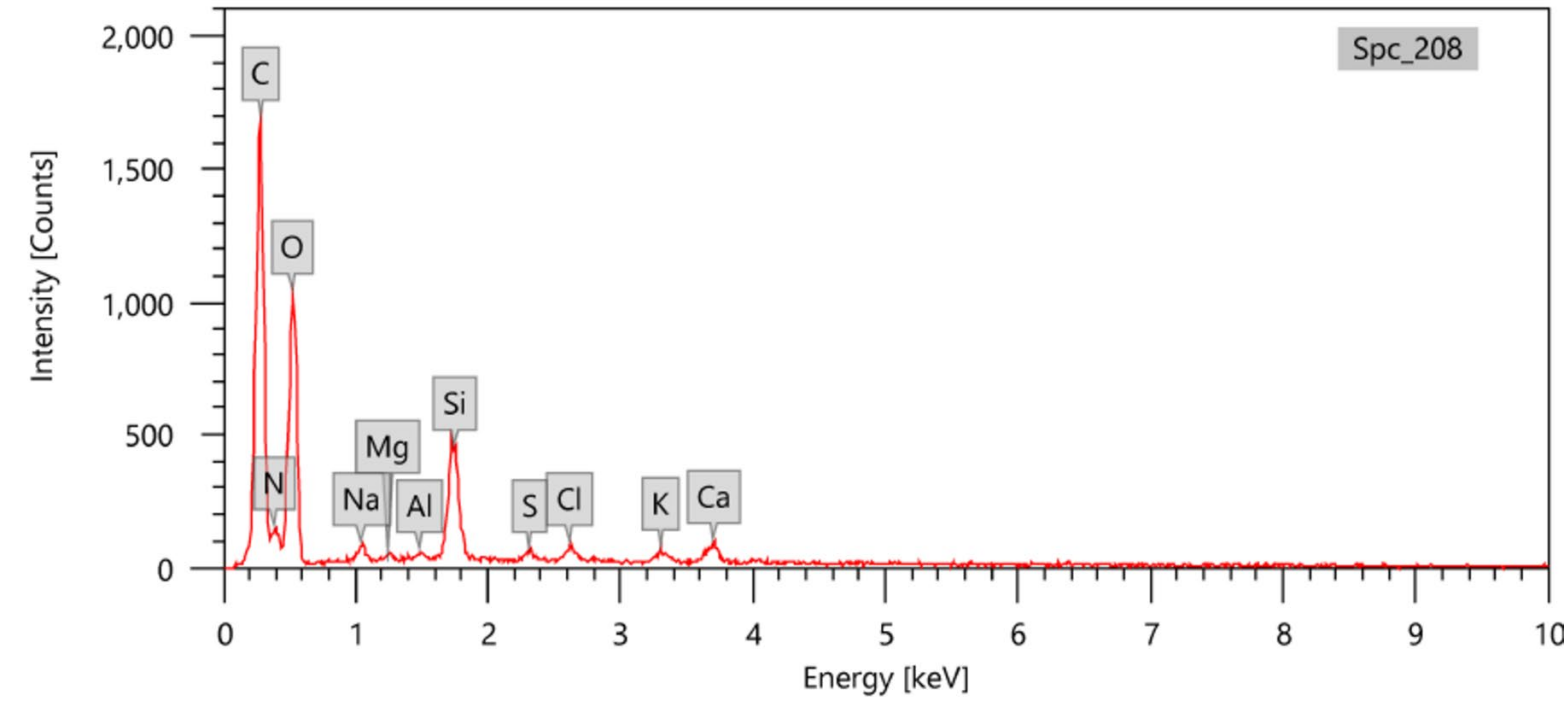
EDX chart for the RS-An sample.

**Figure 7:**
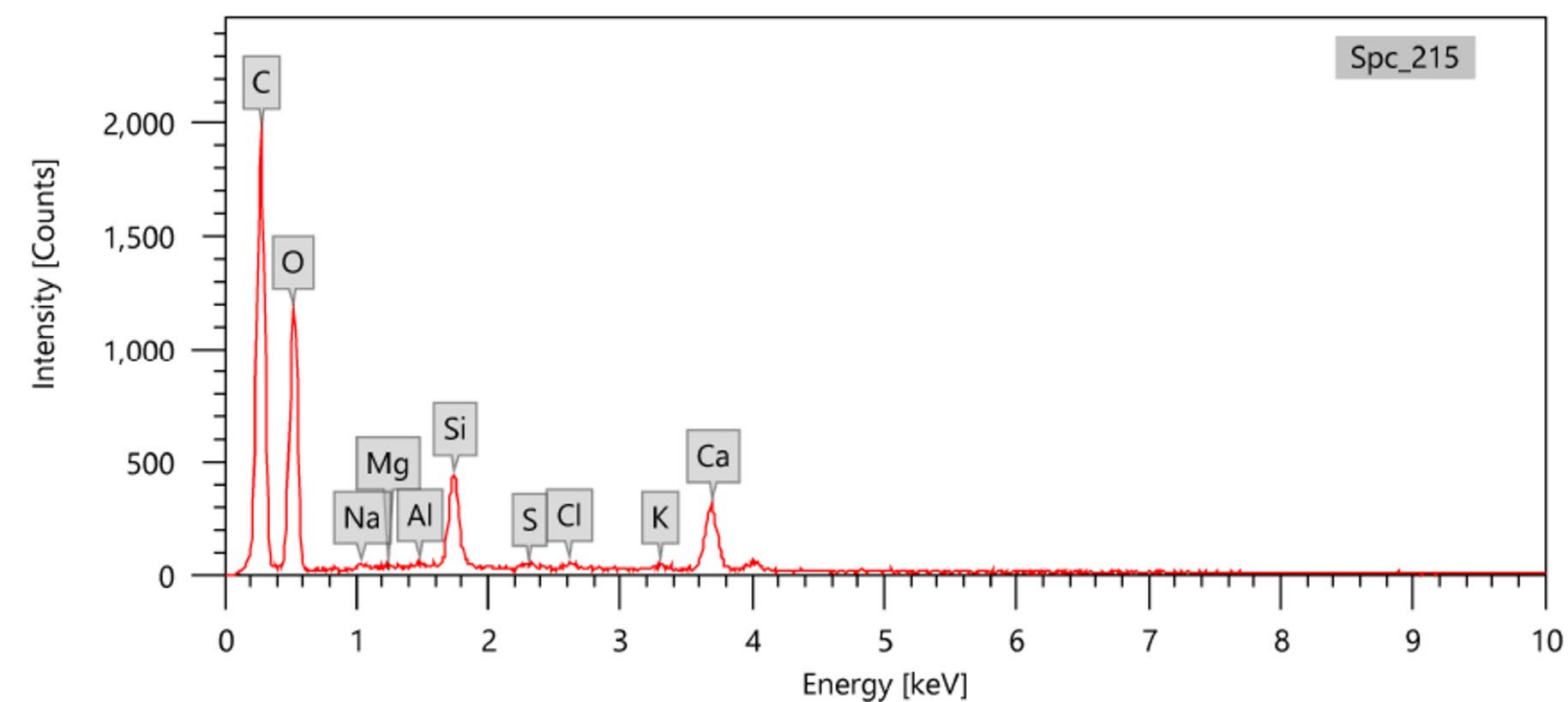
EDX chart for the RS-PVA_C_ sample.

The composition of the prepared samples was analyzed using an Energy Dispersive X-ray (EDX) spectrometer, and the results have been documented in Table 2. As shown in Fig. 6, the presence of nitrogen in the RS-An sample with a percentage of 13.99% and its absence in the RS-PVA_C_ sample. Furthermore, Fig. 7 shows that the percentage of calcium in RS-PVA_C_ was found to be lower than that in RS-An.

### Scanning electron microscope

**Figure 8:**
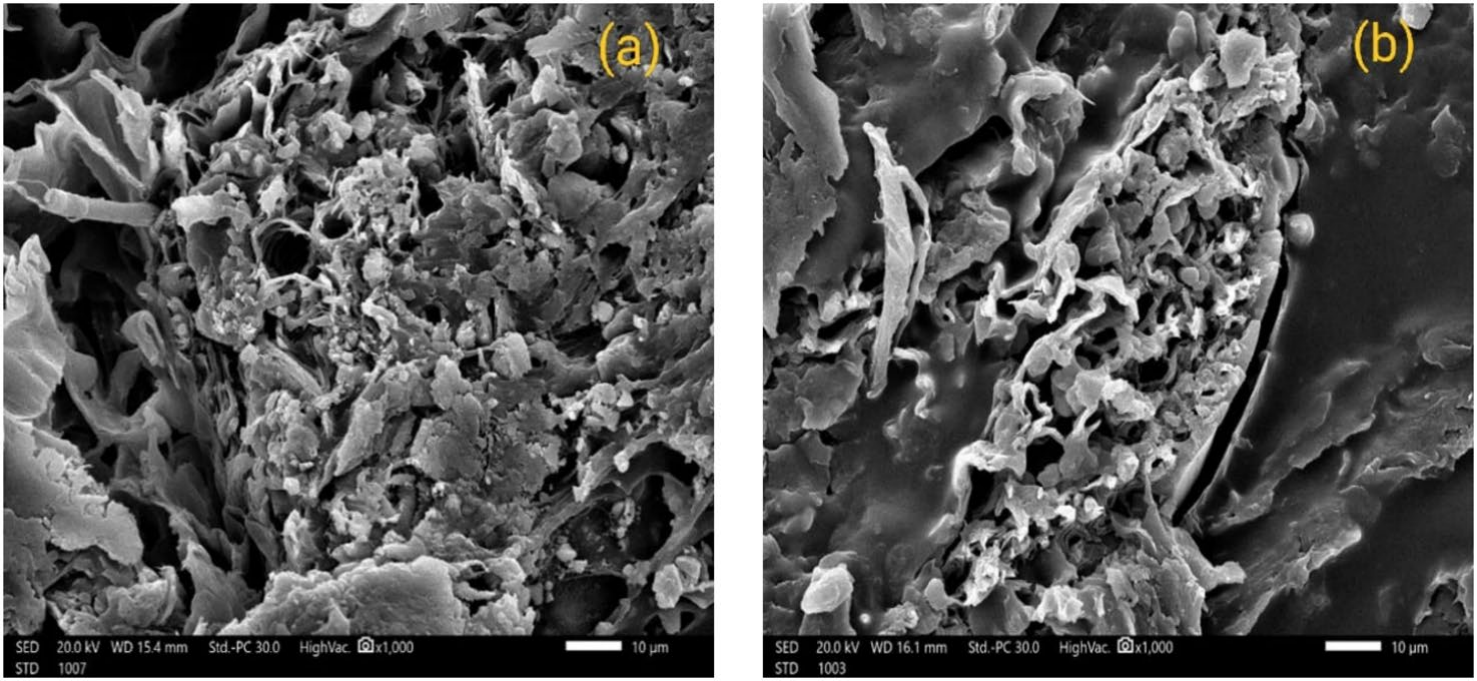
SEM images of (**a**) RS-PVA_C_ and (**b**) RS-An samples.

The morphology of the prepared samples was analyzed by SEM to study their microstructure. SEM–EDX of the selected samples gave microanalysis data on their qualitative and semi-quantitative chemical composition. Based on the SEM micrograph, it is evident that the pores are placed very closely to each other, resulting in a decrease in photon transmission. Therefore, it leads to attenuating the photons within the randomly distributed particles, further impeding their propagation^42^. Figure 8 depicts the clear visibility of the pores of rice straw, with image 8-a showing that the pores of the straw were significantly filled and covered with polyvinyl acetate glue. Conversely, in the sample containing animal glue, image 8-b indicated that animal glue covered the pores to a lesser extent. This can be attributed to the higher viscosity of PVA_C_ glue than animal glue. Furthermore, it confirmed the excellent mixing of the samples.

### Mechanical testing

**Figure 9:**
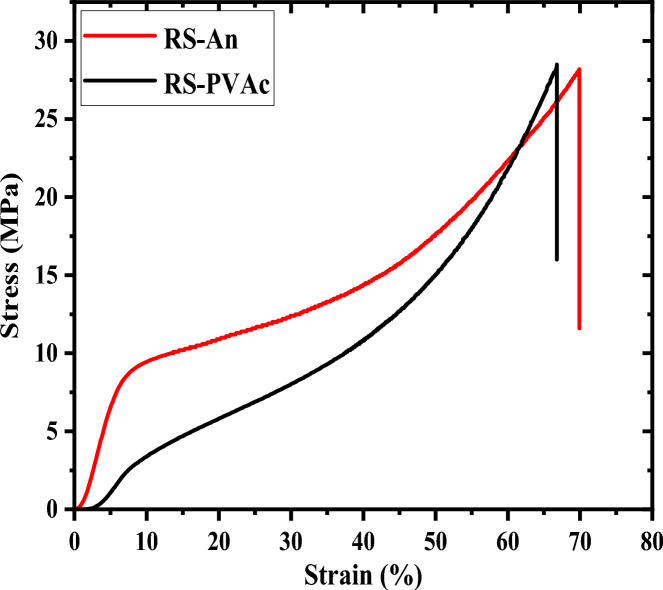
Stress–strain curves of (**a**) RS-An and (**b**) RS-PVA_C_.

Figure 9 depicts the stress–strain curves for RS-An and RS-PVA_C_ samples. As seen in Fig. 9, the RS-PVA_C_ sample endured an applied force of 28.5 MPa before reaching its breaking point. In comparison, the RS-An sample reaches its breaking point at an applied load of approximately 28.2 MPa^43^.

### Shielding parameters

The MACs in the energy region between 0.059 and 1.408 MeV were measured experimentally, theoretically using XCOM software, and by Geant4 simulation code. The comparison between experimental, theoretical, and Geant4 simulation results for the investigated samples is presented in Table 3. The relative deviation (Δ%) between the experimental and XCOM values of MACs at the examined photon energies varies between 0.10 and 2.99%. This close agreement validates the experimental technique using a narrow beam and thin absorber with the theoretical values. Furthermore, the relative deviation (δ%) between the XCOM and Geant4 simulation values of MACs and the relative deviation (RD%) between the experimental and Geant4 values of MACs showed good agreement at all the selected energies. The results demonstrated that the simulated values of the MACs using the Geant4 code are very close to those resulting from the XCOM database and experimental measurements.

**Table 3:** Comparison between the experimental, XCOM, and Geant4 values of the MACs of the prepared samples.

Energy (MeV)	MAC (cm^2^/g)											
	RS-An						RS-PVA_C_					
	XCOM	EXP	Δ%	Geant4	δ%	RD%	XCOM	EXP	Δ%	Geant4	δ%	RD%
59.53	0.1811	0.1777	− 1.83	0.1783	− 1.50	0.33	0.1904	0.1888	− 0.80	0.1870	− 1.77	− 0.95
80.99	0.1602	0.1565	− 2.28	0.1589	− 0.79	1.53	0.1638	0.1651	0.83	0.1624	− 0.82	− 1.63
121.78	0.1419	0.1406	− 0.90	0.1406	− 0.86	0	0.1429	0.1441	0.89	0.1418	− 0.69	− 1.59
244.69	0.1142	0.1125	− 1.45	0.1135	− 0.58	0.88	0.1143	0.1108	− 2.99	0.1134	− 0.78	2.34
344.28	0.1009	0.1002	− 0.62	0.1004	− 0.47	0.19	0.1009	0.1003	− 0.57	0.1009	0.04	0.59
661.66	0.0770	0.0765	− 0.63	0.0774	0.49	1.17	0.0770	0.0775	0.63	0.0775	0.63	0
778.90	0.0715	0.0701	− 1.91	0.0719	0.54	2.56	0.0715	0.0729	1.92	0.0720	0.70	− 1.23
964.13	0.0646	0.0647	0.10	0.0650	0.56	0.46	0.0647	0.0648	0.20	0.0650	0.54	0.30
1173.23	0.0587	0.0583	− 0.57	0.0588	0.24	0.97	0.0587	0.0590	0.45	0.0588	0.09	− 0.33
1332.50	0.0550	0.0546	− 0.70	0.0552	0.34	1.09	0.0550	0.0544	− 1.12	0.0550	0.10	1.10
1408.01	0.0534	0.0524	− 1.85	0.0536	0.32	2.29	0.0535	0.0533	− 0.31	0.0536	0.19	0.56

**Figure 10:**
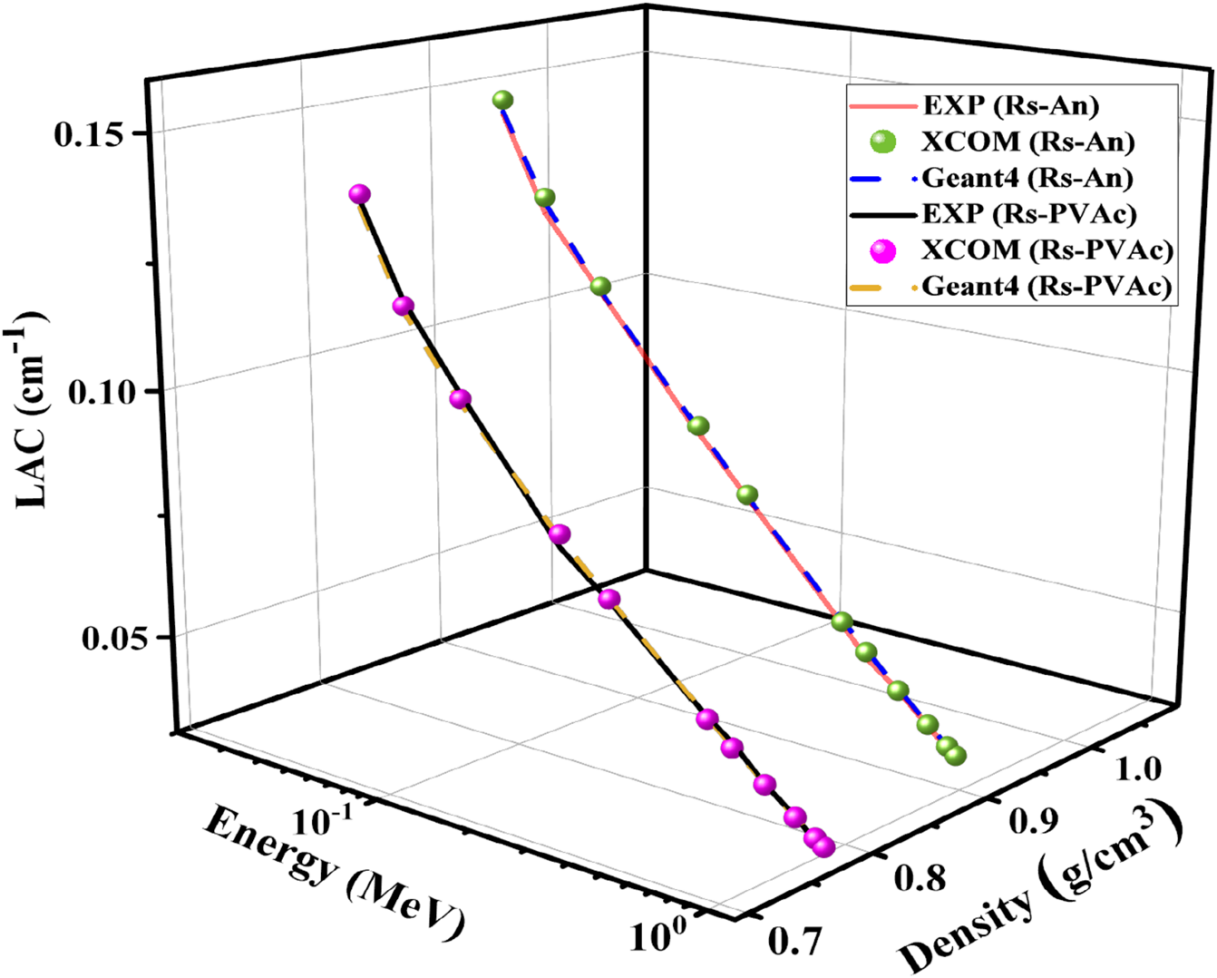
LAC of experimental, theoretical, and Geant4 simulated values of RS-An and RS-PVA_C_ samples.

Figure 10 shows the experimental, theoretical, and Geant4 simulated values of LAC with photon energy. It is evident that there is a good agreement between the experimental, theoretical, and simulation results. Figure 10 shows that the LAC values decrease as energy increases. The maximum value of LAC at lower energy (0.059 MeV) is 0.1507 cm^−1^, which belongs to RS-An, and 0.1395 cm^−1^ for the RS-PVA_C_ sample. Also, LAC is 0.0649 cm^−1^ and 0.0573 cm^−1^ at moderate energy (0.6617 MeV), and at higher energy (1.408 MeV), is 0.0445 cm^−1^ and 0.0394 cm^−1^ for the RS-An and RS-PVA_C_ samples, respectively. It is observed that at photon energies ranging from 0.059 to 0.121 MeV, the LAC decreases sharply as the photon energy increases in this range. This is because at energies lower than 0.125 MeV, the cross-sections for the photoelectric interactions are sufficiently high, which depends on Z^4^/E^3.5^^44^, where Z is the atomic number of the absorbing element, and E is the photon energy. Moreover, as the photon energy increases to exceed 0.121 MeV, the LAC of each composite slightly decreases with increasing photon energy. This is because at this intermediate energy range, the effect of photoelectric absorption decreases, and Compton scattering becomes the dominant mechanism. As expected, the higher the gamma-ray energies, the higher the penetration properties due to the dominance of the pair production process above 1.22 MeV. Figure 10 demonstrates that the RS-An composite is better at attenuating gamma rays than the RS-PVA_C_ composite.

**Figure 11:**
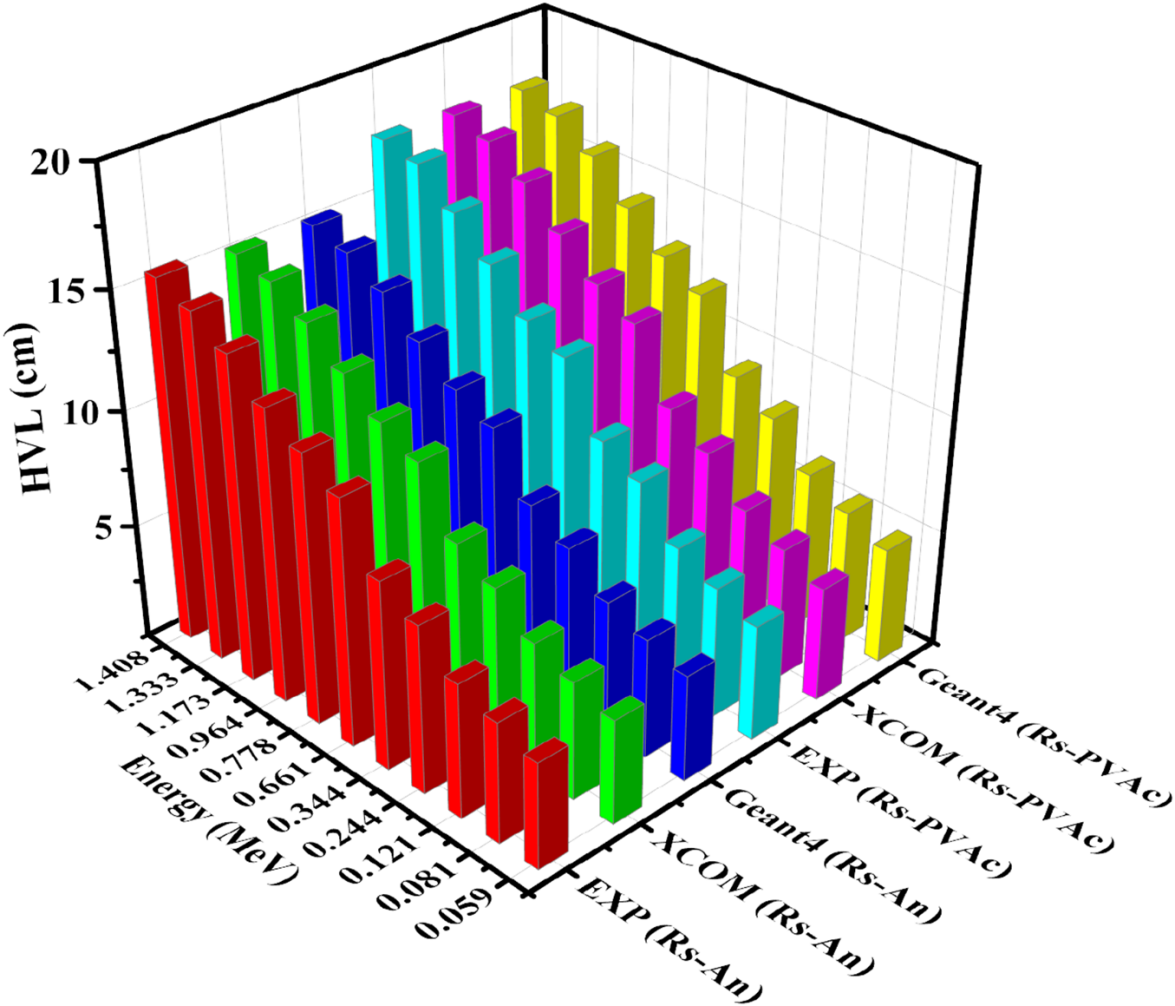
HVL of experimental, theoretical, and Geant4 simulated values of RS-An and RS-PVA_C_ samples.

**Figure 12:**
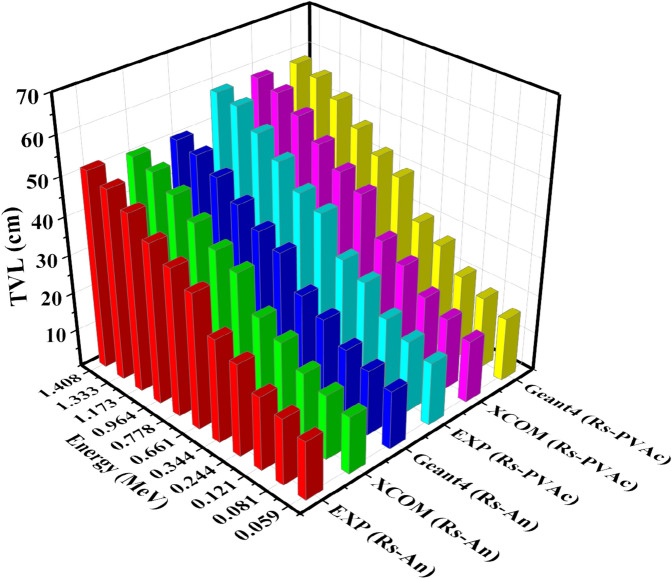
TVL of experimental, theoretical, and Geant4 simulated values of RS-An and RS-PVA_C_ samples.

HVL and TVL parameters are more practically used to describe a material’s shielding capability against photons and for designing practical shields. HVL is the thickness of the samples required to reduce incident photon intensity by one-half. The shielding effectiveness of the sample is inversely proportional to its HVL value. In Fig. 11, the results of HVL values plotted against photon energies show that HVL values increase with increasing photon energies. The experimental results revealed that HVL decreased from 4.968, 12.096, and 17.592 cm for RS-PVAC to 4.599, 10.680, and 15.576 cm for RS-An samples at photon energies of 0.059, 0.6617, and 1.408 MeV, respectively. Also, Fig. 11 shows the agreement between XCOM values and simulated values by Geant4 code. Similarly, Fig. 12 displays the investigated samples’ TVL experimental, theoretical, and Geant4 values. It is worth noting that there is a commendable concurrence between the Geant4 simulated data and the theoretical data. Figure 12 indicates that the TVL values rise as the energy increases, highlighting the greater efficacy of the RS-An sample over the RS-PVA_C_ sample.

**Figure 13:**
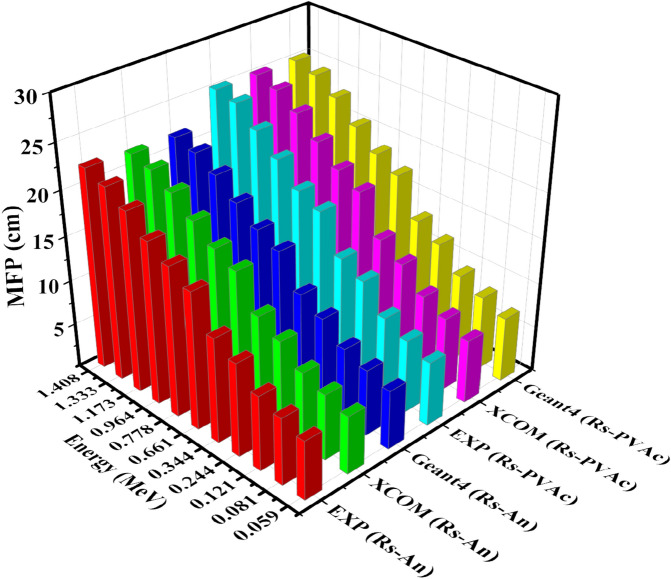
MFP of experimental, theoretical, and Geant4 simulated values of RS-An and RS-PVA_C_ samples.

Figure 13 illustrates the variation of the experimental, theoretical, and Geant4 simulated values of MFP of the studied samples among different photon energies. The MFP values increase as gamma-ray energy increases. When the MFP values are low, there is a higher likelihood that gamma rays will be absorbed or weakened as they travel through a medium^45^. Obviously, in the RS-An sample, the photon loses its energy at a shorter distance than in the RS-PVA_C_ sample. For all studied models, it can also be noted that there is a close match between the experimental, theoretical, and Geant4 code results.

**Figure 14:**
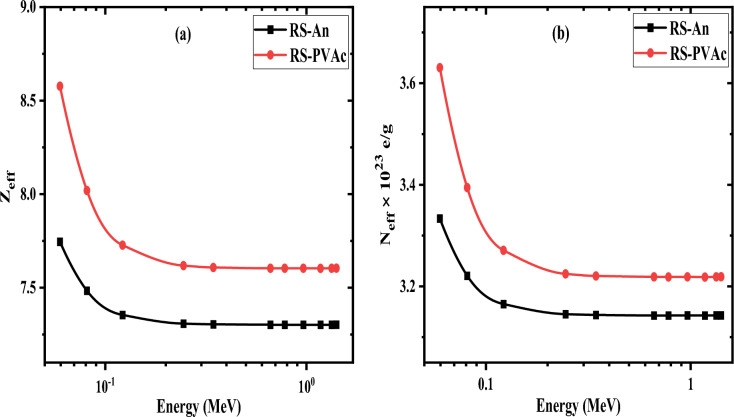
The variation of (**a**) Z_eff_ and (**b**) N_eff_ of the RS-An and RS-PVA_C_ samples at different energies.

The Z_eff_ of the present samples was calculated from the determined MACs. The obtained results are displayed graphically in Fig. 14. As can be seen from Fig. 14a, Z_eff_ is a function of the elemental composition of the sample, so the values of Z_eff_ increased as the concentration of heavy elements increased. Figure 14a indicates that the Z_eff_ values vary from 7.60 to 8.58 for the RS-PVA_C_ and from 7.30 to 7.75 for the RS-An sample, as energy varies from 0.059 to 1.408 MeV. From Fig. 14a, it is obvious that the Z_eff_ of RS-PVA_C_ is comparatively higher than that of the RS-An sample. This is increasing mainly due to the higher concentration of element Ca^46^ in the RS-PVA_C_ sample. For RS-PVA_C_ and RS-An samples, the relation of the N_eff_ values with the photon energy in the range of 0.059–1.408 MeV is presented in Fig. 14b. One can notice that the variations in N_eff_ values are very similar to the trends identified for Z_eff_ values. N_eff_ values vary at 0.059 MeV from 6.53 × 10^25^ to 7.34 × 10^25^ electron /g and at 1.408 MeV from 6.15 × 10^25^ to 6.51 × 10^25^ electron/g for RS-An and RS-PVA_C_ samples, respectively.

**Figure 15:**
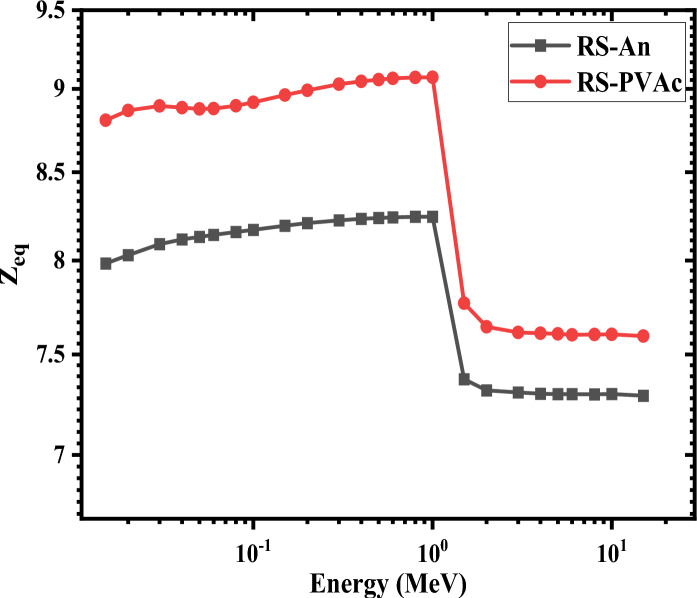
Z_eq_ of RS-An and RS-PVA_C_ samples at different energies.

The Z_eq_ describes the shielding characteristics of the chosen polymers pertaining to equivalent elements and is also considered when determining the buildup factors. The best shielding materials are those with higher Z_eq_. The Z_eq_ values of the RS-An and RS-PVA_C_ samples as a function of photon energy in the range of 0.015–15 MeV are depicted in Figure 15. As can be seen in Fig. 15, the RS-An sample has the lowest Z_eq_ values, whereas the RS-PVA_C_ sample has the highest values. Furthermore, it is also apparent that at 1 MeV, the Z_eq_ reaches its maximum value for all composites. This behavior can be illustrated due to the Compton scattering (CS) interactions that dominate in the mid-(γ) energy region. As the Z_eq_ calculations mostly depended on the ratio of (MAC_CS_/MAC_total_), there is likely significant CS in the medium energy zone, which accounts for the greater reported rise in Z_eq_ values. The pair production process then takes over in the higher energy areas, causing Z_eq_ to drop rapidly as the γ-ray energy approaches 1.22 MeV.

**Figure 16:**
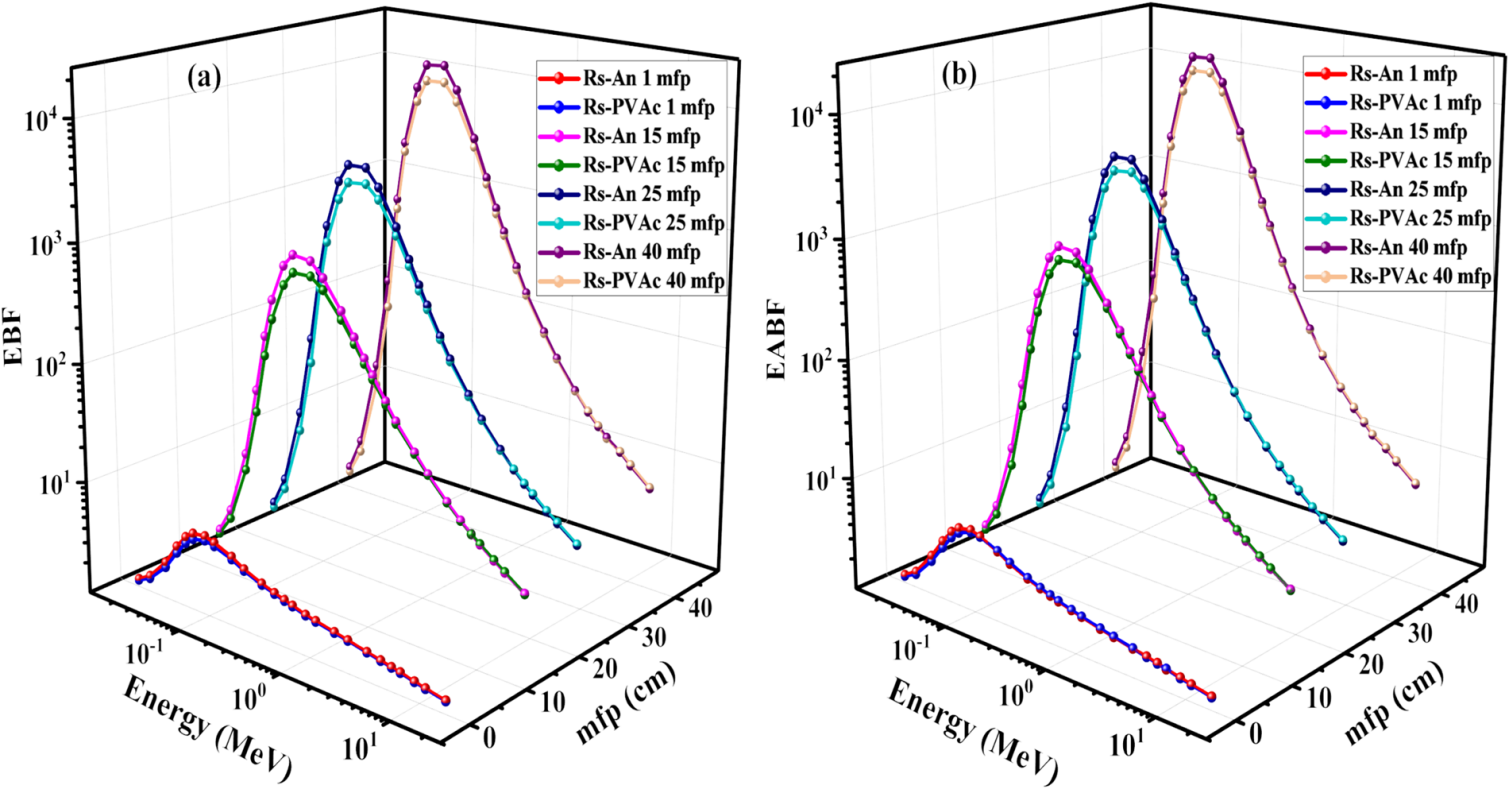
The values of (**a**) EBF and (**b**) EABF for RS-An and RS-PVA_C_ samples as a function of energy at penetration depths of 1, 15, 25, and 40 mfp.

The dependence of the buildup factors (BFs) of the investigated samples with photon energy between 0.015 and 15 MeV are graphically plotted in Fig. 16 at fixed penetration depth values of 1, 15, 25, and 40 mfp. Figure 16a and b can be separated into three energy regions: the first is the photoelectric absorption region, which is considered the main interaction mechanism at low energies, where BFs of the samples have small values in this energy region. The second region is due to the Compton scattering, which is the predominant interaction at the intermediate energies. In this intermediate energy region, EBF and EABF values increase rapidly with increasing the photon energies until reaching their maximum value. This trend occurs since the γ-ray photons are not completely extracted, but their energies are reduced. These photons existed in the sample for a long time, causing multiple Compton scattering interactions, which raised the EBF and EABF values to higher values. The third region is due to the pair production mechanism, and it is the main interaction process at high energies. Figure 16a presents that EBF maximum values are dependent on deep penetration and the composition of materials^47^. EBF values increase until they reach their maximum value and then decrease with increasing energy. Similarly, Fig. 16b shows the same trend for the variation of EABF values with photon energy. This can be discussed in the fundamentals of partial interaction processes. At low energies, EBF and EABF values are the lowest because photons are absorbed at high energies. At intermediate ranging, EBF and EABF values were the largest because photons were degrading by scattering in medium^44,48^. Moreover, the highest BFs values were observed at a deep penetration of 40 mfp while the lowest values were obtained at 1 mfp.

The effectiveness of the current work’s shielding materials was compared to commonly used materials for gamma ray shielding, including polymer clay, pure high-density polyethylene, recycled high-density polyethylene, poly boron, natural bentonite, and nylon 6. The comparison, presented in Table 4, revealed that the current work’s materials exhibited promising attenuation parameters, particularly at low and intermediate energies. These findings highlight the potential of the current work’s materials as efficient and environmentally friendly options for gamma ray shielding.

**Table 4:** Comparison of the present work with other previously reported shielding materials.

Shielding material	Density (g/cm^3^)	Energy (MeV)			References
		0.0595	0.6616	1.3325	
High density polyethylene	0.944	0.188	0.087	0.063	^48^
Poly boron	0.971	–	0.084	0.059	^49^
Recycle high density polyethylene	0.995	0.213	0.098	0.066	^50^
Natural bentonite	0.850	–	0.067	0.048	^51^
Unsaturated polyester and nano clay	1.210	–	0.089	0.040	^52^
Naylon 6	1.140	–	0.098	0.069	^53^
RS-An	0.847	0.150	0.064	0.046	This work
RS-PVA_C_	0.738	0.139	0.057	0.040	

## Conclusion

In the current study, two new composites based on a mixture of rice straw, either with animal glue or polyvinyl acetate glue, were prepared and examined as a plentiful and low-cost matrix to be used in radiation shielding applications. The SEM was utilized to study the morphology and distribution of rice straw within the glue. The SEM analysis revealed that the rice straw is mixed and distributed well within either animal glue or polyvinyl acetate glue. The mechanical test revealed that the stress and strain of the RS-An sample were 25.2 MPa and 69.9%, respectively. On the other hand, the RS-PVA_C_ sample showed values of approximately 25.5 MPa and 66.8% for stress and strain, respectively. The MACs of the prepared samples were calculated experimentally and compared to those obtained theoretically using the XCOM program. This comparison proves a good agreement between the experimental and theoretical data. The results demonstrated that the LAC for the RS-An sample was higher than that of the RS-PVA_C_ sample. The HVL and TVL values of RS-An are less than those of RS-PVA_C_. These results reveal that the RS-An sample is promising for practical applications. Furthermore, the Geant4 Monte Carlo simulation code was also employed to validate the experimental results. The simulated outcomes generated by the Geant4 program were compared to the theoretical results obtained from the XCOM program. It was observed that the Geant4 code accurately simulated real-world data, resulting in a reasonably strong correlation between the practical and theoretical results. Finally, all measurements conducted in this study clearly demonstrate that the rice straw composites serve as a crucial and environmentally friendly solution for radiation protection. The findings highlight the potential of these composites to effectively shield against radiation, contributing to sustainable practices in the field of radiation protection.

## Data Availability

All data generated or analyzed during this study are included in this published article.

## References

[1] Alabsy, M. T., Abbas, M. I., El-khatib, A. Y. & El-Khatib, A. M. Attenuation properties of poly methyl methacrylate reinforced with micro/nano ZrO_2_ as gamma-ray shields. *Sci. Rep.* **14**, 1–18 (2024). 38218742 10.1038/s41598-024-51551-4 PMC10787785

[2] Samdani, M. *et al.* Gamma shielding performance of B_2_O_3_/BaO-based glassy system: Synthesis and simulation study. *Radiat. Phys. Chem.* **214**, 111301 (2024). 10.1016/j.radphyschem.2023.111301

[3] Chaturvedi, A. & Jain, V. Effect of Ionizing radiation on human health. *Int. J. Plant Environ.* **5**, 200–205 (2019). 10.18811/ijpen.v5i03.8

[4] Basha, B. *et al.* Synthesis of Bi_2_O_3_ doping powder from CRT-screen waste glass: Physical, structural, and radiation attenuation properties. *Radiat. Phys. Chem.* **214**, 111279 (2024). 10.1016/j.radphyschem.2023.111279

[5] Katubi, K. M. *et al.* Radiation Shielding efficiency of lead-tungsten-boron glasses with Sb, Al, and Bi against gamma, neutron and charge particles. *Appl. Radiat. Isot.* **204**, 111139 (2024). 38104471 10.1016/j.apradiso.2023.111139

[6] Cao, D., Yang, G., Bourham, M. & Moneghan, D. Gamma radiation shielding properties of poly (methyl methacrylate)/Bi_2_O_3_ composites. *Nucl. Eng. Technol.* **52**, 2613–2619 (2020). 10.1016/j.net.2020.04.026

[7] Ban, C. C. *et al.* Modern heavyweight concrete shielding: Principles, industrial applications and future challenges; review. *J. Build. Eng.* **39**, 102290 (2021). 10.1016/j.jobe.2021.102290

[8] Kaboorani, A. & Riedl, B. Improving performance of polyvinyl acetate (PVA) as a binder for wood by combination with melamine based adhesives. *Int. J. Adhes. Adhes.* **31**, 605–611 (2011). 10.1016/j.ijadhadh.2011.06.007

[9] Schellmann, N. C. Animal glues: A review of their key properties relevant to conservation. *Stud. Conserv.* **52**, 55–66 (2007). 10.1179/sic.2007.52.Supplement-1.55

[10] Miao, Z. C. *et al.* Preparation of bone glue adhesives using epichlorohydrin modification. *Adv. Mater. Res.* **557**, 1005–1008 (2012). 10.4028/www.scientific.net/AMR.557-559.1005

[11] Kaboorani, A. & Riedl, B. Mechanical Performance of Polyvinyl Acetate (PVA)-Based Biocomposites. In *Biocomposites: Design and Mechanical Performance* (Elsevier Ltd., 2015). 10.1016/B978-1-78242-373-7.00009-3.

[12] Rahman, M. R., Islam, M. N., Huque, M. M., Hamdan, S. & Ahmed, A. S. Effect of chemical treatment on rice husk (RH) reinforced polyethylene (PE) composites. *BioResources* **5**, 854–869 (2010). 10.15376/biores.5.2.854-869

[13] Guan, Y. *et al.* Biomass molded fuel in China: Current status, policies and suggestions. *Sci. Total Environ.* **724**, 138345 (2020). 32408467 10.1016/j.scitotenv.2020.138345

[14] Khalid, N., Ahmad, S., Kiani, S. N. & Ahmed, J. Removal of lead from aqueous solutions using rice husk. *Sep. Sci. Technol.* **33**, 2349–2362 (1998). 10.1080/01496399808545279

[15] Ozioko, F. U. Effect of carbonization temperature on wear rate behaviour of rice husk ash reinforced epoxy composites. *Leonardo Electron. J. Pract. Technol.* **10**, 172–182 (2011).

[16] Atuanya, C. U., Olaitan, S. A., Akagu, C. C., Onukwuli, O. D. & Menkiti, M. C. Effect of rice husk filler on mechanical properties of polyethylene matrix composite. *Int. J. Curr. Res. Rev.* **5**, 111 (2013).

[17] Ranjan, C. *et al.* Fabrication and strength analysis of rice straw fibers reinforced epoxy biodegradable composite. *Mater. Today Proc.* **46**, 331–335 (2021). 10.1016/j.matpr.2020.08.299

[18] Pachla, E. C., Silva, D. B., Stein, K. J., Marangon, E. & Chong, W. Sustainable application of rice husk and rice straw in cellular concrete composites. *Constr. Build. Mater.* **283**, 122770 (2021). 10.1016/j.conbuildmat.2021.122770

[19] Elhussieny, A. *et al.* Valorisation of shrimp and rice straw waste into food packaging applications. *Ain Shams Eng. J.* **11**, 1219–1226 (2020). 10.1016/j.asej.2020.01.008

[20] Taha, M. *et al.* Unveiling the potential of rice straw nanofiber-reinforced HDPE for biomedical applications: Investigating mechanical and tribological characteristics. *J. Funct. Biomater.* **14**, 366 (2023). 37504861 10.3390/jfb14070366 PMC10381549

[21] Megahed, M., Ali-Eldin, S. S., Abd El Moezz, S. M. & Abdalla, W. S. Synthesis of developed rice straw sheets and glass fiber-reinforced polyester composites. *J. Compos. Mater.* **54**, 3381–3394 (2020). 10.1177/0021998320915641

[22] Ozturk, M., Karaaslan, M., Akgol, O. & Sevim, U. K. Mechanical and electromagnetic performance of cement based composites containing different replacement levels of ground granulated blast furnace slag, fly ash, silica fume and rice husk ash. *Cem. Concr. Res.* **136**, 106177 (2020). 10.1016/j.cemconres.2020.106177

[23] El-Kassas, A. M. & Elsheikh, A. H. A new eco-friendly mechanical technique for production of rice straw fibers for medium density fiberboards manufacturing. *Int. J. Environ. Sci. Technol.* **18**, 979–988 (2021). 10.1007/s13762-020-02886-8

[24] Xu, H. *et al.* A new process of preparing rice straw-reinforced LLDPE composite. *Polymers* **14**, 2243 (2022). 35683917 10.3390/polym14112243 PMC9183130

[25] El-Sayed, T. A. Performance of heavy weight concrete incorporating recycled rice straw ash as radiation shielding material. *Prog. Nucl. Energy* **135**, 103693 (2021). 10.1016/j.pnucene.2021.103693

[26] Mostafa, A. G. Studies on the shielding properties of transparent glasses prepared from rice husk silica. *Am. J. Mod. Phys.s* **4**, 149 (2015). 10.11648/j.ajmp.20150404.11

[27] El-Khatib, A. M. *et al.* Novel slag/natural rubber composite as flexible material for protecting workers against radiation hazards. *Sci. Rep.* **13**, 1–17 (2023). 37608066 10.1038/s41598-023-40846-7 PMC10444829

[28] Abd-Elzaher, M., Badawi, M. S., El-Khatib, A. & Thabet, A. A. Determination of full energy peak efficiency of NaI(Tl) detector depending on efficiency transfer principle for conversion form experimental values. *World J. Nucl. Sci. Technol.* **02**, 65–72 (2012). 10.4236/wjnst.2012.23011

[29] Dumpala, M. Lead nitrate loaded, novel clay-based bricks as radiation shielding materials for building applications. *Radiat. Prot. Environ.* **42**, 47 (2019). 10.4103/rpe.RPE_36_18

[30] Sagon, S. & Surujpaul, P. P. The use of local alternative materials as structural shielding for diagnostic radiological facilities. *J. Glob. Radiol.* **6**, e1050 (2020). 10.7191/jgr.2020.1050

[31] Gouda, M. M., El-Khatib, A. M., Abbas, M. I., Al-Balawi, S. M. & Alabsy, M. T. Gamma attenuation features of white cement mortars reinforced by micro/nano Bi_2_O_3_ particles. *Materials* **16**, 1580 (2023). 36837210 10.3390/ma16041580 PMC9966324

[32] El-Khatib, A. M. *et al.* A new environmentally friendly mortar from cement, waste marble and nano iron slag as radiation shielding. *Materials* **16**, 2541 (2023). 37048835 10.3390/ma16072541 PMC10095434

[33] Rammah, Y. S., Ali, A. A., El-Mallawany, R. & El-Agawany, F. I. Fabrication, physical, optical characteristics and gamma-ray competence of novel bismo-borate glasses doped with Yb2O3 rare earth. *Phys. B* **583**, 412055 (2020). 10.1016/j.physb.2020.412055

[34] Hosseini, M. A., Malekie, S. & Kazemi, F. Experimental evaluation of gamma radiation shielding characteristics of polyvinyl alcohol/tungsten oxide composite: A comparison study of micro and nano sizes of the fillers. *Nucl. Instrum. Methods Phys. Res. Sect. A* **1026**, 166214 (2022). 10.1016/j.nima.2021.166214

[35] Almisned, G. *et al.* Novel Cu/Zn reinforced polymer composites: Experimental characterization for radiation protection efficiency (rpe) and shielding properties for alpha, proton, neutron, and gamma radiations. *Polymers* **13**, 3157 (2021). 34578058 10.3390/polym13183157 PMC8473252

[36] Alabsy, M. T., Gouda, M. M., Abbas, M. I., Al-Balawi, S. M. & El-Khatib, A. M. Enhancing the gamma-radiation-shielding properties of gypsum–lime–waste marble mortars by incorporating micro-and nano-PbO particles. *Materials* **16**, 1577 (2023). 36837205 10.3390/ma16041577 PMC9966484

[37] Alzahrani, F. M. A., Albarkaty, K. S., Çalişkan, F., Olarinoye, I. O. & Al-Buriahi, M. S. Physical, microstructural, and radiation energy absorption properties of recycled CRT-screen glass doped with Bi_2_O_3_. *J. Radiat. Res. Appl. Sci.* **16**, 100727 (2023).

[38] Trubey, D. K. *New Gamma-Ray Buildup Factor Data for Point Kernel Calculations: Ans-6. 4. 3 Standard Reference Data*. (1988).

[39] El-Khatib, A. M., Abbas, Y. M., Badawi, M. S., Hagag, O. M. & Alabsy, M. T. Gamma radiation shielding properties of recycled polyvinyl chloride composites reinforced with micro/nano-structured PbO and CuO particles. *Phys. Scr.* **96**, 125316 (2021). 10.1088/1402-4896/ac35c3

[40] Allison, J. *et al.* Recent developments in Geant4. *Nucl. Instrum. Methods Phys. Res. Sect. A: Accel. Spectrom. Detect. Assoc. Equip.* **835**, 186–225 (2016). 10.1016/j.nima.2016.06.125

[41] Alabsy, M. T. & Elzaher, M. A. Radiation shielding performance of metal oxides/EPDM rubber composites using Geant4 simulation and computational study. *Sci. Rep.* **13**, 7744 (2023). 37173378 10.1038/s41598-023-34615-9 PMC10182101

[42] Mishra, S. P., Garnayak, S., Bhuyan, R. K. & Nath, G. Design and analysis of effective graded microwave absorbing material for low observable technology. *Indian J. Pure Appl. Phys.* **58**, 629–634 (2020).

[43] Sunardi, *et al.* Particleboard characterization using sawdust from sengon wood, mahogany wood, bayur wood, and rice husk ash as composite fillers. *IOP Conf. Ser. Mater. Sci. Eng.* **909**, 1–11 (2020). 10.1088/1757-899X/909/1/012028

[44] Abbas, M. I. *et al.* Gamma-ray attenuation and exposure buildup factor of novel polymers in shielding using geant4 simulation. *Materials* **14**, 5051 (2021). 34501139 10.3390/ma14175051 PMC8434600

[45] Katubi, K. M. *et al.* Radiation attenuation and optical properties of P_2_O_5_-based glass system. *J. Radiat. Res. Appl. Sci.* **16**, 100688 (2023).

[46] Kaçal, M. R., Akman, F. & Sayyed, M. I. Evaluation of gamma-ray and neutron attenuation properties of some polymers. *Nucl. Eng. Technol.* **51**, 818–824 (2019). 10.1016/j.net.2018.11.011

[47] Ravangvong, S. *et al.* Build-up factors and fast neutron properties of some plastic and polymer for shielding materials: A simulation. *J. Appl. Res. Sci. Technol. (JARST)* **20**, 47–56 (2021).

[48] El-Khatib, A. M. *et al.* Gamma attenuation coefficients of nano cadmium oxide/high density polyethylene composites. *Sci. Rep.* **9**, 16012 (2019). 31690761 10.1038/s41598-019-52220-7 PMC6831599

[49] Biswas, R., Sahadath, H., Mollah, A. S. & Huq, M. F. Calculation of gamma-ray attenuation parameters for locally developed shielding material: Polyboron. *J. Radiat. Res. Appl. Sci.* **9**, 26–34 (2016).

[50] Mahmoud, M. E. *et al.* Recycled high-density polyethylene plastics added with lead oxide nanoparticles as sustainable radiation shielding materials. *J. Clean. Prod.* **176**, 276–287 (2018). 10.1016/j.jclepro.2017.12.100

[51] Hager, I. Z. *et al.* Nano-structured natural bentonite clay coated by polyvinyl alcohol polymer for gamma rays attenuation. *J. Theor. Appl. Phys.* **13**, 141–153 (2019). 10.1007/s40094-019-0332-5

[52] Bagheri, K., Razavi, S. M., Ahmadi, S. J., Kosari, M. & Abolghasemi, H. Thermal resistance, tensile properties, and gamma radiation shielding performance of unsaturated polyester/nanoclay/PbO composites. *Radiat. Phys. Chem.* **146**, 5–10 (2018). 10.1016/j.radphyschem.2017.12.024

[53] More, C. V., Bhosale, R. R. & Pawar, P. P. Detection of new polymer materials as gamma-ray-shielding materials. *Radiat. Eff. Defects Solids* **172**, 469–484 (2017). 10.1080/10420150.2017.1336765

